# Towards the Identification of New Genes Involved in ABA-Dependent Abiotic Stresses Using *Arabidopsis* Suppressor Mutants of *abh1* Hypersensitivity to ABA during Seed Germination

**DOI:** 10.3390/ijms140713403

**Published:** 2013-06-27

**Authors:** Agata Daszkowska-Golec, Edyta Chorazy, Miroslaw Maluszynski, Iwona Szarejko

**Affiliations:** Department of Genetics, Faculty of Biology and Environmental Protection, University of Silesia, Jagiellonska 28, 40-032 Katowice, Poland; E-Mails: edytachorazy@interia.pl (E.C.); miroslaw.maluszynski@us.edu.pl (M.M.); iwona.szarejko@us.edu.pl (I.S.)

**Keywords:** *Arabidopsis*, suppressor mutant, abscisic acid, abiotic stress, map-based cloning

## Abstract

Abscisic acid plays a pivotal role in the abiotic stress response in plants. Although great progress has been achieved explaining the complexity of the stress and ABA signaling cascade, there are still many questions to answer. Mutants are a valuable tool in the identification of new genes or new alleles of already known genes and in elucidating their role in signaling pathways. We applied a suppressor mutation approach in order to find new components of ABA and abiotic stress signaling in Arabidopsis. Using the *abh1* (*ABA hypersensitive 1*) insertional mutant as a parental line for EMS mutagenesis, we selected several mutants with suppressed hypersensitivity to ABA during seed germination. Here, we present the response to ABA and a wide range of abiotic stresses during the seed germination and young seedling development of two suppressor mutants—*soa2* (suppressor of *abh1* hypersensitivity to *ABA 2*) and *soa3* (*suppressor of abh1 hypersensitivity to ABA 3*). Generally, both mutants displayed a suppression of the hypersensitivity of *abh1* to ABA, NaCl and mannitol during germination. Both mutants showed a higher level of tolerance than Columbia-0 (Col-0—the parental line of *abh1*) in high concentrations of glucose. Additionally, *soa2* exhibited better root growth than Col-0 in the presence of high ABA concentrations. *soa2* and *soa3* were drought tolerant and both had about 50% fewer stomata per mm^2^ than the wild-type but the same number as their parental line—*abh1*. Taking into account that suppressor mutants had the same genetic background as their parental line—*abh1*, it was necessary to backcross *abh1* with Landsberg erecta four times for the map-based cloning approach. Mapping populations, derived from the cross of *abh1* in the Landsberg erecta background with each suppressor mutant, were created. Map based cloning in order to identify the suppressor genes is in progress.

## 1. Introduction

### 1.1. The Role of Abscisic Acid in the Seed Germination Process

Seed germination commences with water uptake, which is triphasic and lasts until the emergence of the radicle. A complicated network of changes in transcription and protein metabolism, together with changes in the endogenous content of phytohormones, such as abscisic acid (ABA), gibberellins (GAs), brassinosteroids (BRs), ethylene (ET), salicylic acid (SA) and auxins, regulate the process of seed germination in order to ensure the survival of future generations [1]. The ability to tolerate or resist salt, osmotic, dehydration and cold stress is essential for plant survival. When a sudden abiotic stress occurs between the first exposure of seeds to water and the cotyledon greening, the seed germination process can be arrested [2–4]. In order to maintain or break the period of arrested germination and to complete the germination program, different metabolic pathways, including phytohormone biosynthesis and signaling, are involved. Among them, ABA plays a major function.

ABA signaling and its key role in the adaptation of plants to stress conditions has been the focus of many studies since the early 90’s [5–7]. Together with ABA, gibberellins (GAs) act as a main regulating factor in seed germination; their role is antagonistic compared to ABA. Hormonal balance is under the control of environmental factors. Before imbibition, seeds are in a dormant state or are quiescent when nondormant seeds are considered. According to North *et al*. [8] dormancy is an inability of viable seeds to undergo germination under optimal conditions. Dormancy is induced and maintained by ABA and released by GAs. Before seed imbibition, over 10,000 different mRNAs are stored in dry Arabidopsis seeds [9]. Among them, transcripts of genes with ABA response cis-elements in their promoter sequences, such as ABRE, are overrepresented [10]. This implies a major role of ABA in the seed germination process. During imbibition, light and cold act together to break dormancy by inducing the biosynthesis of GAs. Simultaneously, a decreased level of ABA is observed due to a lower level of the expression of genes encoding ABA biosynthesis enzymes, such as NCED3 (9-cis-epoxycarotenoid dioxygenase) and a higher level of the expression of genes encoding ABA catabolism enzymes, such as CYP707A [11]. Many components of the ABA signal transduction pathway take part in the regulation of seed germination under abiotic stress conditions.

The identification of ABA receptors—PYR/PYL/RCAR (PYRABACTIN RESISTANCE1/PYRABACTIN RESISTANCE 1-LIKE/REGULATORY COMPONENT OF ABA RESPONSE 1) revolutionized the understanding of the interactions between ABA signaling components [12–15]. These receptors regulate the phosphatases PP2C (PROTEIN PHOSPHATASES 2 C). Phosphatases PP2Cs are thought to be co-receptors of ABA [13,15–18]. However, since ABA is buried deep in the pocket of PYR/PYL/RCAR and there is no direct contact between ABA and PP2Cs, the co-receptor concept deviates from that of the classical sense in which two proteins bind the ligand [17]. PP2Cs interact with SnRK2s (SUCROSE NON FERMENTING 1 RELATED KINASES 2) and inhibit their action in the absence of ABA. SnRK2s are important for activating the transcription factors that are crucial for seed germination, such as ABI5 (ABA INSENSITIVE 5) [19–21]. ABI5, ABI3 (ABA INSENSITIVE 3) and ABI4 (ABA INSENSITIVE 4) belong to three transcription factor families, which contain the domains: B3-(ABI3), APETALA2-(ABI4), and bZIP-(ABI5) and regulate seed-specific and/or ABA related gene expression [5,22–25]. Several mutants in *ABI3*, *ABI4* and *ABI5* genes were identified, initially based on their ABA-insensitive phenotype during seed germination; however, it was discovered that they also displayed defects in the response to glucose, NaCl and osmotic stress during germination and seedling development [26–29]. The function of other important regulators of seed germination in response to ABA and abiotic stresses was also recognized using ABA mutants insensitive and hypersensitive to ABA during seed germination. The first group includes mutants carrying defects in genes encoding phosphatases, such as *ABI1* (*ABA INSENSITIVE 1*) [30–32], *ABI2* (*ABA INSENSITIVE 2*) [30,31], transcription factors, such as *CHO1* (*CHOTTO1*) [33] or components of ubiquitination machinery*: RHA2* (*RING H2*) [34] and *AIRP1* (*ABA INSENSITIVE RING PROTEIN 1*) [35]. In contrast, mutations that led to a hypersensitivity to ABA were identified in the gene-encoding protein phosphatase 2C (AtPP2CA)-*AHG3* (*ABA HYPERSENSITIVE GERMINATION 3*) [36], a gene encoding poly(A)-specific ribonuclease (PARN)-*AHG2* (*ABA HYPERSENSITIVE GERMINATION 2*) [37] and in genes related to RNA metabolism, such as *SAD1* (*SUPERSENSITIVE TO ABA AND DROUGHT 1*) [38], *HYL1* (*HYPONASTIC LEAVES 1*) [39] and *ABH1* (*ABA HYPERSENSITIVE 1*) [40].

ABA is thought to be the main stress hormone acting at seed germination and early seedling development. However, both processes under stress conditions are also regulated by other phytohormones as was revealed through the use of mutants in genes involved in phytohormone metabolism and signaling pathways [1].

### 1.2. Suppressor Mutants as a Tool in the Identification of New Genes/Alleles and Interactions between Them and the Other Components of the Pathway of Interest

Suppressor screens have been used successfully in Arabidopsis and other model organisms in order to discover new genes or the interactions between those that are already known in signaling pathways. The creation of suppressor mutants has its origin in the mutagenesis of a parental line, which is a mutant that carries a mutation in a gene with an unknown or not fully elucidated function. Among mutation techniques, chemical mutagenesis with EMS (Ethyl methanesulfonate) [41–45] or MNU (*N*-nitroso-*N*-methylurea) [46] has been most commonly used, while physical mutagenesis (γ rays) was rarely used [47,48]. Suppressor mutants can be classified into two groups: intra- and extragenic supressors according to the position of the suppressor mutation, within or outside the mutated gene. The first group includes mutants that carry mutations within the same gene as in the parental mutant line. This type of mutant was identified, for example, by Belkhadir *et al* [41] in the studies on deciphering the role of the extracellular domain of BRI1 (BRASSINOSTEROID INSENSITIVE 1), which acts as brassinosteroid receptor. Seeds of a *bri1-5* mutant that carried a mutation in the region of the *BRI1* encoding leucine rich repeat (LRR) of the extracellular domain were mutagenized with EMS. *bri1-5* exhibited a lower level of the BRI1 peptide than the wild-type. Molecular analysis revealed that the BRI1 protein was not properly folded in the mutant and thus it was only localized in the endoplasmic reticulum, not in the plasma membrane like in the wild-type. The intragenic suppressor mutation *bri1-5R1* was identified in the same region of *BRI1* as the primary mutation *bri1-5*. Analysis of the suppressor mutant showed that the suppressor mutation was able to revert the level of BRI1 protein to almost the same level as in the wild-type and the peptide was localized properly in the plasma membrane. Using the suppressor mutant, Belkhadir *et al.* [41] showed the crucial function of the extracellular domain of BRI1 in the localization of the brassinosteroid receptor in the plasma membrane.

The second group of suppressor mutants (extragenic mutants) carry a mutation in a gene other than those present in the parental line. Extragenic mutants prevail among studies engaging suppressor mutations. Extragenic suppressor mutants have been used for the further investigation of gene functions and for deciphering components of signaling pathways, including phytohormones signaling [44,47,48] abiotic stress response [49], biotic stress response [42,43], photosynthesis [50] or morphogenesis [51]. Sugliani *et al*. [48] isolated a suppressor mutant of the *abi3-5* form—the *sua* (*suppressor of abi3-5*), which displayed a hypersensitivity to ABA during germination, in contrast to the phenotype of its parental line—*abi3-5*. The identified suppression was allele-specific, there was no phenotype change in the background of the other *abi3* mutants. It was proven that in the wild-type, two types of *ABI3* transcripts can be produced—*α-ABI3*, which was a full-length transcript, and *β-ABI3*, which was a truncated and nonfunctional mRNA. The regulation of alternative splicing was dependent on the stage of development; the truncated form was produced during the last phase of seed maturation. This type of regulation may have a role in accelerating the time necessary to reduce the level of the *ABI3* transcript just before the initiation of germination. In the case of the *abi3-5* mutant, the *β-ABI3* was the functional transcript because of a frame shift that was caused by the *abi3-5* mutation. Analysis of a *sua* mutant revealed that *SUA* encoded a splicing factor that decreased the level of the *β-ABI3* isoform. A mutation in *SUA* in the *abi3-5* background led to an abnormal ratio of both isoforms and, as a consequence, to an increased sensitivity to ABA during seed germination.

The selection of suppressor mutants and their further phenotypic and molecular analysis is a very important tool in the identification of new genes/alleles, their interactions and in the detection of other components of the pathways of interest. Studies performed by Brady *et al* [52] clearly confirmed the usefulness of suppressor mutants in revealing the components of ABA signaling. They mutagenized seeds of the ABA-hypersensitive mutant—*era1* (*enhanced response to ABA 1*). As a result of selection in the presence of 0.3 μM ABA, the individuals that displayed an insensitivity to ABA during seed germination in contrast to their parental line *era1* were isolated. A series of crosses between the suppressor forms and the already known mutants in ABA-related genes were performed. One of the analyzed suppressors was allelic to the *abi3* mutant. A comparative analysis of the *ABI3* genomic sequence between the suppressor and the wild-type confirmed that the suppressor carried a point mutation in the *ABI3* gene. This result together with very detailed phenotype analysis of the suppressor mutant allowed a working model of interaction between ERA1 and ABI3 to be established. Further analysis revealed that ERA1 is able to farnesylate ABI3 [52].

### 1.3. The Aim of the Present Study

Although the pathway of ABA signaling in regards to plant response to abiotic stresses has been supplemented with new reports in recent years, there are still many gaps in fully understanding this process. Among the genes whose roles have not been fully explained to date is an *ABH1* (*CBP80*) (*ABA hypersensitive 1* (*Cap Binding Protein 80*)) gene. It encodes a large subunit of the nuclear cap binding complex (CBC).

The role of *ABH1* (*CBP80*) continues to be elusive during seed germination in the presence of ABA and in drought response, except for the known phenotype of the *abh1* mutant. Hugouvieux *et al*. [40,53] showed that *abh1* displays a drought-tolerant phenotype. It is able to close its stomata very quickly in response to a water deficit and therefore optimizes the efficiency of water use during stress conditions. Similar trends related to the involvement of *ABH1* (CBP80) in drought response were observed in the potato RNAi line [54]. The Arabidopsis *abh1* mutant is hypersensitive to low ABA concentrations (0.4 μM) [40] and as further analysis revealed also to salt, mannitol, glucose and ACC during seed germination and early seedling development [55]. *abh1* also displays morphological differences when compared to its wild-type Col-0—it has serrated leaves [40] and exhibited a 50% fewer number of stomata [55]. The molecular role of CBC has been well established in relation to its involvement in splicing. It has been shown that CBC binds to the monomethylated cap (GpppN) structure of all RNAs transcribed by RNA polymerase II and participates in the processing of polymerase II RNA primary transcripts. As recent findings have shown, CBC is also involved in pri-miRNA maturation [56–59].

It has been reported that CBP20 and CBP80 (ABH1) are necessary for the ABA-dependent induction of miR159 during seed germination. Positive regulators of the ABA signaling transcription factors, MYB33 and MYB101, are downregulated by miR159. The significantly lower level of mature miR159 in *abh1* and the subsequent accumulation of MYB33 and MYB101 transcripts result in ABA hypersensitivity during germination [58]. MIR159 expression is regulated by ABI3, and partially by ABI5, in the presence of ABA [60]. Daszkowska-Golec *et al*. [55] showed that ABI4 is also possibly engaged in that signalosome. The action of miR159 during seed germination shows a connection between CBP80 (ABH1) and well-known ABA signaling components such as ABI3, ABI5 and ABI4.

Taking into account that CBC is involved in many aspects of the ABA signaling network, there is still a need to explain the exact role of ABH1 (CBP80) and to identify new components that interact with ABH1 (CBP80) in the signaling pathway.

In order to gain insight into the mechanism of ABH1 (CBP80) action in ABA and abiotic stress signal transduction during seed germination, a genetic screen for suppressors of *abh1* during seed germination in the presence of ABA was undertaken. Suppressor mutants were generated after chemical mutagenesis with EMS and selected as insensitive to 0.4 μM ABA which inhibited the germination of its parental line—*abh1* [55,61]. The aim of the present study was to characterize the phenotypic similarities and differences between *abh1* and its suppressor mutants during a wide range of abiotic stresses. Here, we present the detailed characteristics of two suppressor mutants of *abh1: soa2* (*suppressor of abh1 hypersensitivity to ABA* 2) and *soa3* (*suppressor of abh1 hypersensitivity to ABA* 3) in the presence of ABA, ACC and abiotic stresses during seed germination, seedling development and the mature stage, as a first step in identifying the suppressor genes.

## 2. Results

### 2.1. *soa2* and *soa3* Exhibit the Suppression of *abh1* Hypersensitivity to 0.4 μM ABA during Seed Germination

**Figure 1:**
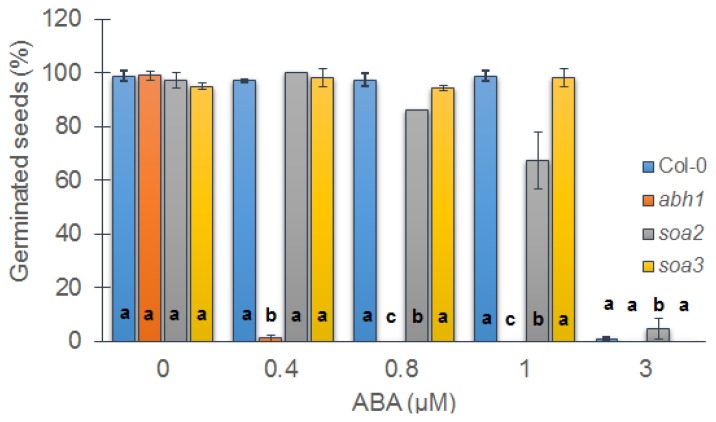
Response of Col-0, *abh1*, *soa2* and *soa3* to different ABA concentrations during seed germination. Values represent the mean ± SD of three biological replicates; 100–200 seeds of each genotype were analyzed in each. For each concentration means followed by the same letter do not differ significantly according to Fisher’s projected LSD (*p* ≤ 0.05).

Suppressor mutants of *abh1*—*soa2* (*suppressor of abh1 hypersensitivity to ABA* 2) and *soa3* (*suppressor of abh1 hypersensitivity to ABA* 3) were isolated based on their insensitivity to 0.4 μM ABA during seed germination after chemical mutagenesis of an *abh1* mutant with EMS. In the presence of 0.4 μM ABA seed germination of the parental line of suppressor mutants—*abh1* was totally inhibited. All experiments described in the presented study were performed using suppressor mutants in *abh1* background in order to compare responses and find the differences between single *abh1* mutant and the double mutant carrying the *abh1* mutation and the suppressor mutation. In order to establish the response of suppressor mutants to ABA in comparison to *abh1* and Columbia-0 (Col-0—the parental line of *abh1*), a seed germination assay with increasing ABA concentrations was performed. It was shown that both suppressor mutants were capable of germinating not only at 0.4 μM ABA, but also in the presence of higher ABA concentrations, such as 0.8 and 1 μM ABA. However, none of them germinated at a higher level of ABA than Col-0. A concentration of 3 μM ABA completely inhibited the germination of all of the analyzed genotypes (Figure 1).

### 2.2. Both Suppressor Mutants Are Recessive in Relation to *abh1* Hypersensitivity to ABA during Seed Germination and Are Not Allelic

*soa2* and *soa3* were backcrossed to the original mutant *abh1* and the response of F_1_ and F_2_ progeny to ABA during seed germination was analyzed. The F_1_ progeny of both crosses exhibited a hypersensitivity to 0.4 μM ABA, thus indicating that both suppressor genes (*soa2* and *soa3*) were recessive in relation to *abh1.* This observation was confirmed by an analysis of germination in the F_2_ progeny, which displayed a segregation ratio of 3:1 of ABA hypersensitive to ABA insensitive germinating seeds, respectively (χ^2^_3:1_ = 0.025 and χ^2^_3:1_ = 0.163, *p* ≤ 0.05; Table 1). In both cases, the only segregating gene in the F_2_ progeny was the suppressor gene because both suppressor mutants—*soa2* and *soa3* and their parental line, *abh1*—carried the homozygous mutation in the *ABH1* gene.

In order to test whether *soa2* and *soa3* carried mutations in the same gene or in different loci, an allelism test was performed. *soa2* and *soa3* were crossed to each other and the response of F_1_ and F_2_ progeny was tested in the same manner as previously described. The F_1_ progeny displayed an insensitivity to 0.4 μM ABA. When the F_2_ progeny was analyzed, two phenotypic classes were extracted in a segregation ratio of 11:5 of ABA insensitive to ABA hypersensitive individuals, respectively (*soa2* × *soa3* χ^2^_11:5_ = 1.2 and *soa3* × *soa2* χ^2^_3:1_ = 1.1, *p* ≤ 0.05; Table 2).

A detailed description of the phenotypes and genotypes of plants in each cross are presented in Table 3.

### 2.3. *soa2* and *soa3* Differ in Root Growth in the Presence of High ABA Concentrations

**Figure 2:**
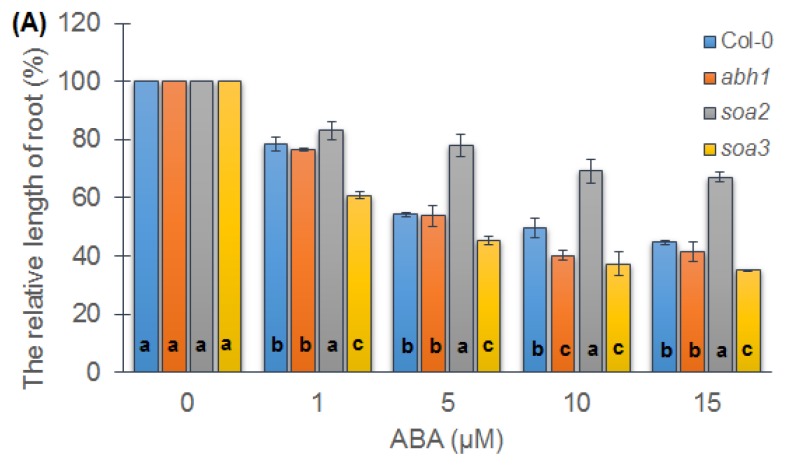

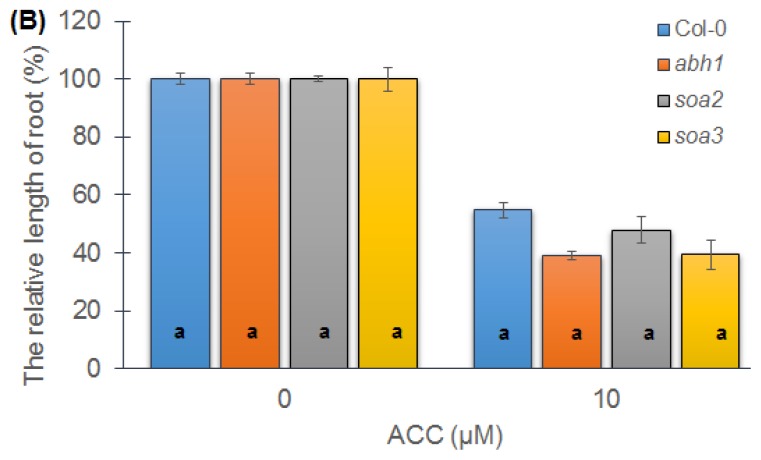
Relative root growth in the presence of different ABA concentrations. Values represent the mean ± SD of three biological replicates; 70–100 seedlings of each genotype were analyzed in each. (**A**) For each concentration, means followed by the same letter do not differ significantly according to Fisher’s projected LSD (*p* ≤ 0.05). Relative root growth is expressed as the % of root growth on the control medium; (**B**) Relative root growth in the presence of ACC. Values represent the mean ± SD of three biological replicates; 70–100 seedlings of each genotype were analyzed in each. For each concentration means followed by the same letter do not differ significantly according to Fisher’s projected LSD (*p* ≤ 0.05). Relative root growth is expressed as the % of root growth on the control medium.

Differences were observed between suppressor mutants during seedling development in relation to root growth in the presence of different ABA concentrations. Only *soa2* displayed an insensitivity to a high ABA concentration when compared to the wild-type Col-0 and *abh1*. In the presence of 15 μM ABA, *soa2* displayed only a 30% reduction in root length when compared to the growth on the control medium, whereas the reduction in root elongation of the wild-type and *abh1* under these conditions was 60% (Figure 2A).

### 2.4. Response of Suppressor Mutants to ACC during Seedling Development

Root growth was analyzed not only in the presence of ABA, but also when a precursor of ethylene, the other phytohormone, was added to the medium. The inhibitory effect of ACC (1-Aminocyclopropane-1-carboxylic acid) on root growth was observed in all of the genotypes studied (Figure 2B). No significant differences between suppressor mutants and *abh1* or Col-0 were detected.

### 2.5. Both Suppressors Are Sensitive to Salt Stress during Seed Germination but Displayed a Differential Response when Young Seedlings Were Tested

**Figure 3:**
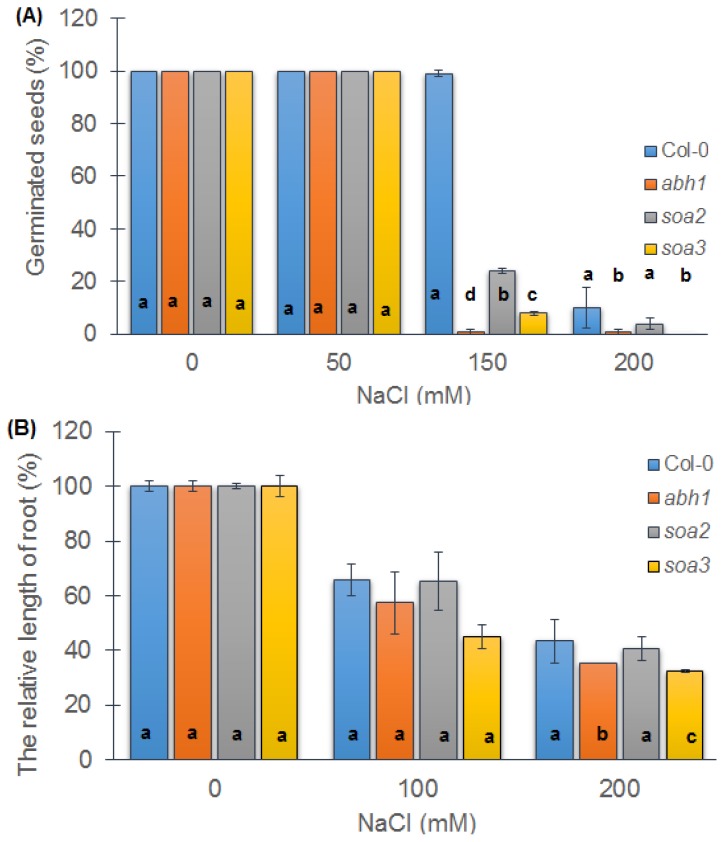
Response of Col-0, *abh1*, *soa2* and *soa3* to different NaCl concentrations during seed germination (**A**) and early seedling development (**B**). (**A**) Values represent the mean ± SD of three biological replicates; 100–200 seeds of each genotype were analyzed in each. For each concentration, means followed by the same letter do not differ significantly according to Fisher’s projected LSD (*p* ≤ 0.05); (**B**) Relative root length of Col-0, *abh1*, *soa2* and *soa3* in the presence of different salt concentrations. Values represent the mean ± SD of three biological replicates; 70–100 seedlings of each genotype were analyzed in each. For each concentration, means followed by the same letter do not differ significantly according to Fisher’s projected LSD (*p* ≤ 0.05). Relative root growth is expressed as the % of root growth on the control medium.

When the response to salt stress was evaluated during seed germination, both suppressors were more tolerant to 150 mM NaCl concentrations than their parental line, *abh1*, but much less tolerant than the wild-type Col-0. There was a strong tendency toward a decreased germination potential when a higher concentration of NaCl such as 200 mM was applied (Figure 3A). The correlation between seed germination of suppressor mutants in the presence of high NaCl and high ABA concentration was observed.

In addition, the *soa2* and *soa3* sensitivity to salt treatment was tested during root elongation. No significant differences among the genotypes studied were observed during development in the presence of 100 mM NaCl. In the presence of a higher NaCl concentration (200 mM), both suppressors displayed a higher reduction of root growth than Col-0 and *abh1*, but the differences between all of the genotypes were small (Figure 3B).

### 2.6. Response to Osmotic Stress during Seed Germination and Seedling Development of Both Mutants Indicate Differences between Suppressors, the Wild-Type and Parental Line, *abh1*

**Figure 4:**
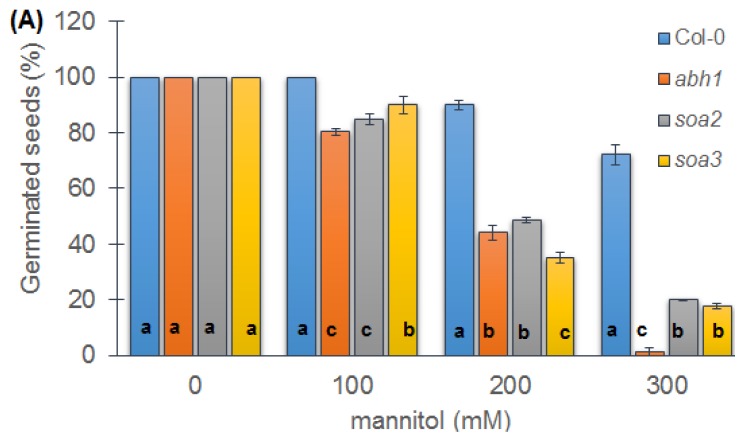

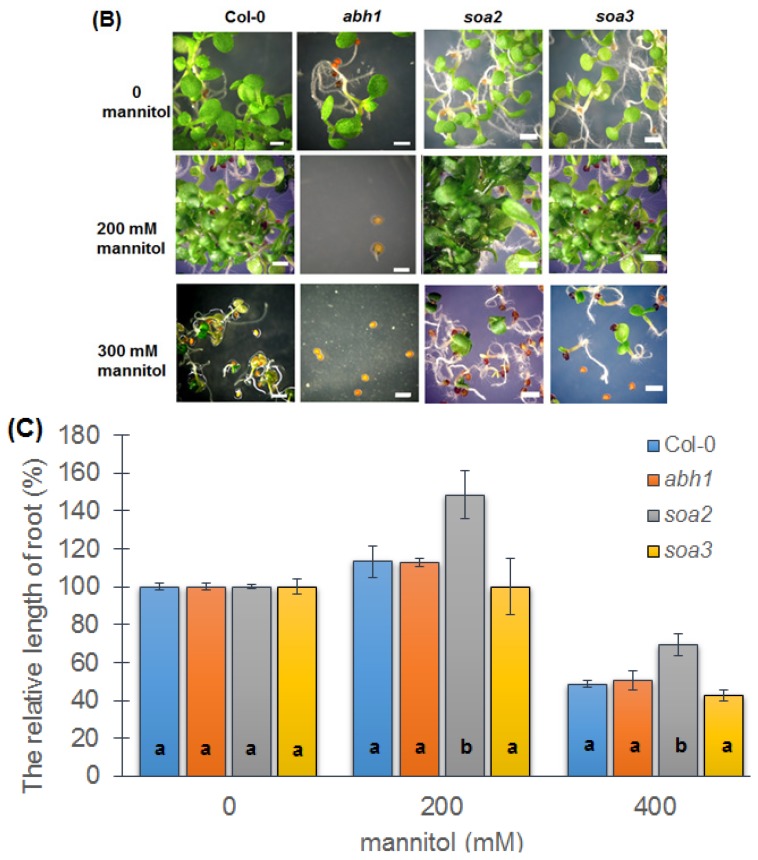
Response of Col-0, *abh1*, *soa2* and *soa3* to different mannitol concentrations during seed germination. (**A**) Values represent the mean ± SD of three biological replicates; 100–200 seeds of each genotype were analyzed in each. For each concentration, means followed by the same letter do not differ significantly according to Fisher’s projected LSD (*p* ≤ 0.05); (**B**) Response of Col-0, *abh1*, *soa2* and *soa3* to different mannitol concentrations during seedling development—greening assay. Photographs were taken at 14th day of development; (**C**) Relative root length of Col-0, *abh1*, *soa2* and *soa3* in the presence of different mannitol concentrations. Values represent the mean ± SD of three biological replicates; 70–100 seedlings of each genotype were analyzed in each. For each concentration, means followed by the same letter do not differ significantly according to Fisher’s projected LSD (*p* ≤ 0.05). Relative root growth is expressed as the % of root growth on the control medium.

In order to check whether the response to salt was salt-specific or universal for the osmotic stress, the response of both suppressors to mannitol as an osmotic factor was examined. It was observed that 60% and 100% of *abh1* seeds showed an inhibition of germination in the presence of 200 and 300 mM of mannitol, respectively. In the case of Col-0, a higher fraction of germinated seeds was observed in the presence of 200 and 300 mM (80% and 70%), when compared to *abh1*. The response of suppressors was intermediate between their parental line, *abh1* and the wild-type, Col-0. In the presence of the highest mannitol concentration, both suppressors were capable of germination at the level of 20%, while *abh1* was not (Figure 4A).

Taking into account that the level of sensitivity to abiotic stresses during germination and early seedling growth should also be determined on the basis of cotyledon development, further analyses were performed on the 14th day after the end of stratification. Both suppressor mutants, *soa2* and *soa3*, showed well-developed and fully green cotyledons in the presence of 200 and 300 mM mannitol (Figure 4B). These results together showed that suppressors displayed a reduced sensitivity to osmotic stress during early seedling development.

Similar to the salt stress, the *soa2* and *soa3* sensitivity to osmotic treatment was also tested during the root elongation growth. *soa2* exhibited a tolerance to osmotic stress by continued root growth on a medium containing 200 mM and 400 mM of mannitol. In the presence of 400 mM, the root elongation was reduced to 40%–50% in the case of Col-0, *abh1* and *soa3* whereas *soa2* showed a 70% root growth compared to control conditions (Figure 4C).

### 2.7. *soa2* and *soa3* Are Insensitive to High Glucose Concentrations

**Figure 5:**
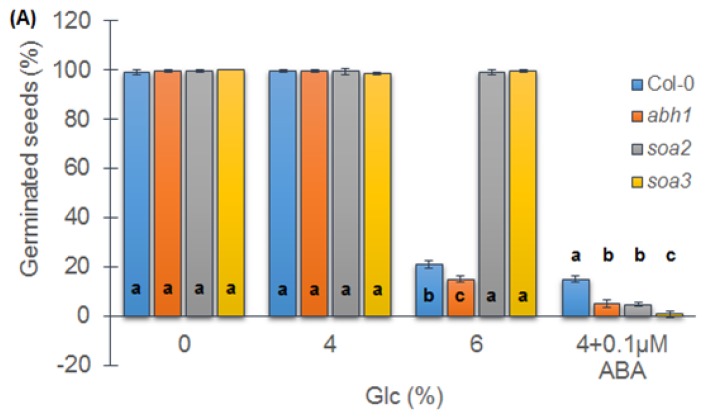

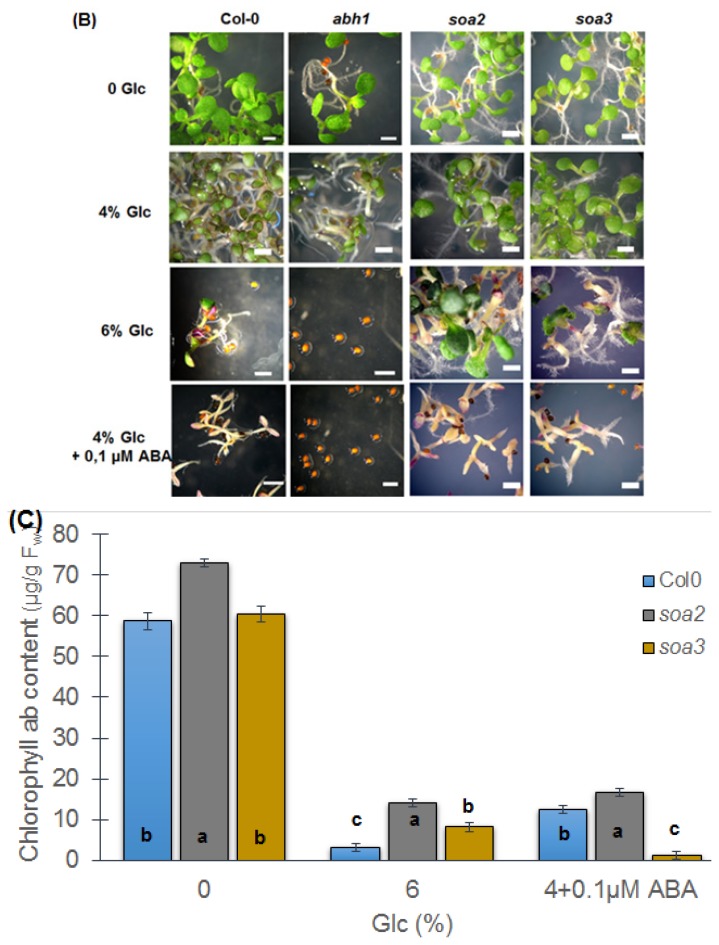
Response of Col-0, *abh1*, *soa2* and *soa3* to different glucose concentrations during seed germination. (**A**) Values represent the mean ± SD of three biological replicates; 100–200 seeds of each genotype were analyzed in each. For each concentration, means followed by the same letter do not differ significantly according to Fisher’s projected LSD (*p* ≤ 0.05); (**B**) Response of Col-0, *abh1*, *soa2* and *soa3* plants to different glucose concentrations during seedling development—greening assay. Photographs were taken at 14th day of development; (**C**) The chlorophyll ab content in Col-0, *soa2* and *soa3* in the presence of different glucose concentrations. Values represent the mean ± SD of three biological replicates; 100–200 seedlings of each genotype were analyzed in each. For each concentration means followed by the same letter do not differ significantly according to Fisher’s projected LSD (*p* ≤ 0.05).

Seed germination of Col-0 is highly affected by high glucose concentration. In order to check whether suppressors displayed a different response to glucose (Glc) than their parental line, *abh1* and the wild-type Col-0, a seed germination assay in the presence of different glucose concentrations was performed. In the presence of 4% of Glc, no differences between tested genotypes were observed. The application of 6% of Glc significantly inhibited seed germination of Col-0 and *abh1*, while both suppressors were able to germinate at the same level as observed on the control medium. When ABA in non-inhibitory concentration (0.1 μM) was added to the medium containing 4% of Glc, a decreased level of seed germination was observed in all of the genotypes studied (Figure 5A). The seedlings developed from seeds that germinated in the presence of 4% Glc with an addition of 0.1 μM ABA exhibited white cotyledons, and inhibited development of roots and were not able to survive (Figure 5B).

In order to quantify the effect of a 6% Glc and 4% of Glc with an addition of 0.1 μM ABA on cotyledon greening, the chlorophyll content of the seedlings was measured. This analysis confirmed the results of the germination test related the tolerance to a high Glc concentration of both suppressor mutants (Figure 5C). Both suppressors displayed a slightly higher level of chlorophyll ab than the wild-type in the presence of 6% Glc. On the medium containing 4% of Glc with an addition of 0.1 μM ABA, a different response of suppressors was observed. The chlorophyll ab content was significantly higher in *soa2* than in *soa3*. Taking together, a higher reduction of seed germination and a lower level of chlorophyll in the developed seedlings in *soa3* mutant indicate its higher sensitivity to low ABA concentrations combined with 4% glucose than the *soa2* mutant.

### 2.8. *soa2* and *soa3* Display a Drought-Tolerant Phenotype

**Figure 6:**
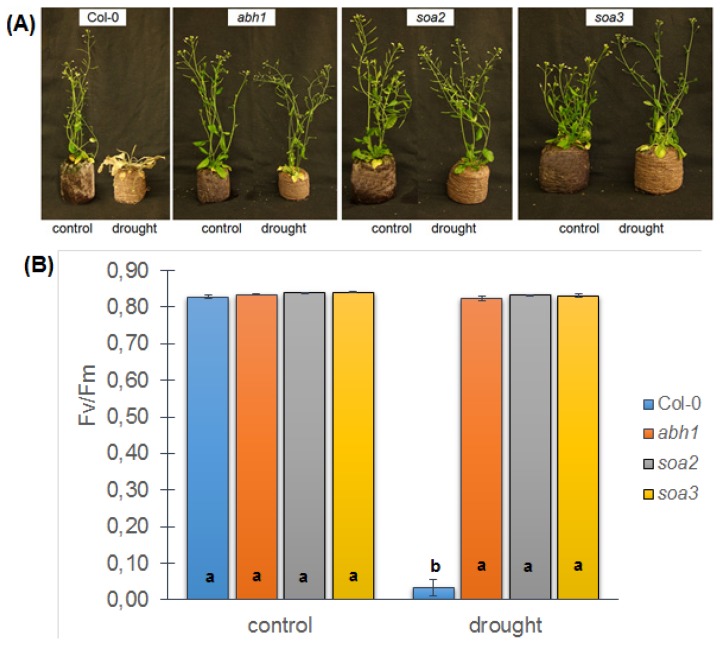

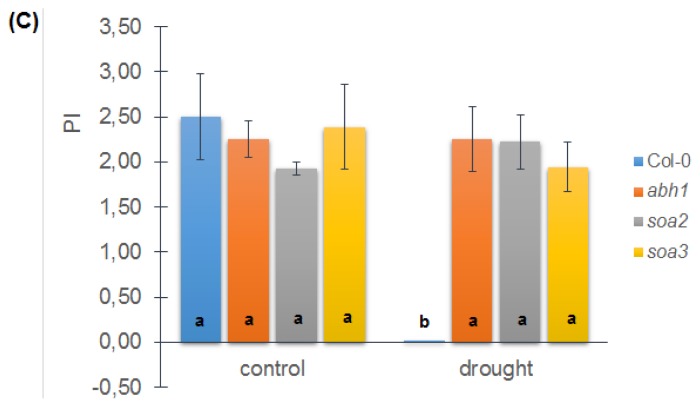
Response of Col-0, *abh1*, *soa2* and *soa3* mature plants to drought. (**A**) Representative plants of Col-0, *abh1*, *soa2* and *soa3*; (**B**) Fluorimetric measurements of *Fv*/*Fm* and (**C**) *PI* parameters on the 30th day of the assay. Values represent the mean ± SD of three biological replicates. For each genotype, means followed by the same letter do not differ significantly according to Fisher’s projected LSD (*p* ≤ 0.05).

The effect of the suppression of *abh1* hypersensitivity to ABA described above was exhibited as the inhibition of the *abh1* phenotype in response to stresses, such as salt, osmotic and exogenous glucose, which were applied during seed germination and early seedling development. To check whether the suppression also acts at other stages of plant development, mature plants of the genotypes studied were exposed to drought stress. The *abh1* mutant is known to be drought tolerant because of its rapid closure of the stomata in stress conditions [40]. To compare the response of Col-0, *abh1* and suppressors to drought stress, drought treatment was applied for 4 weeks, after which the plants were re-watered for 3 days and their phenotype was examined (Figure 6A).

Col-0 did not survive the stress treatment, whereas *abh1* and the suppressor mutants displayed a slightly wilty phenotype after 26 days of drought, but restored a normal phenotype after 3 days of re-watering. Fluorimetric measurements on the 30th day of the experiment were conducted using PocketPea (Hansatech, Norfolk, England). Neither the *abh1* mutant nor its suppressor mutants displayed any drought-induced changes in PSII performance, in contrast to Col-0 (Figure 6A,B).

**Figure 7:**
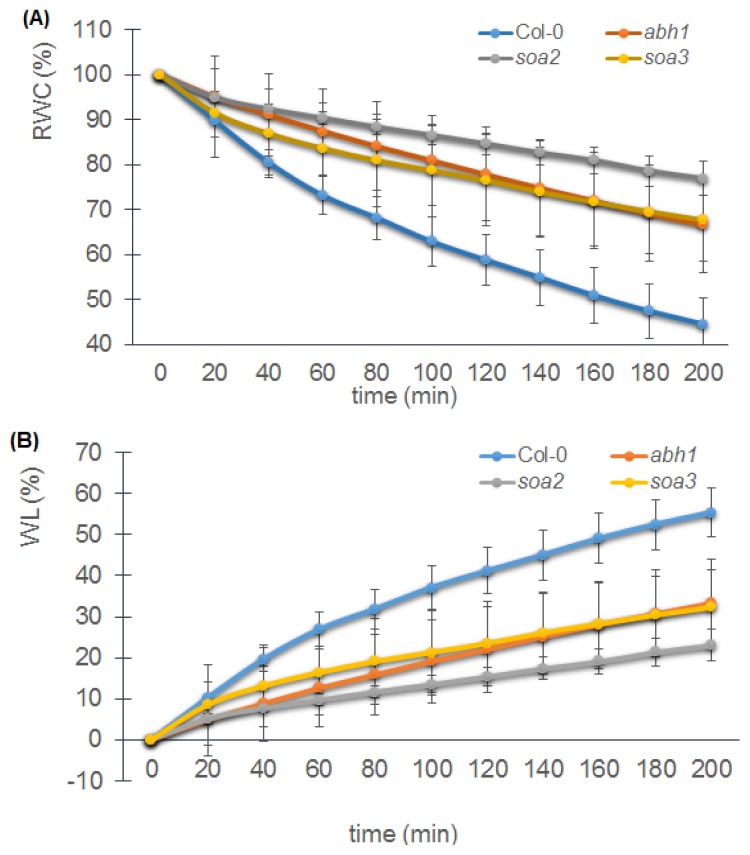

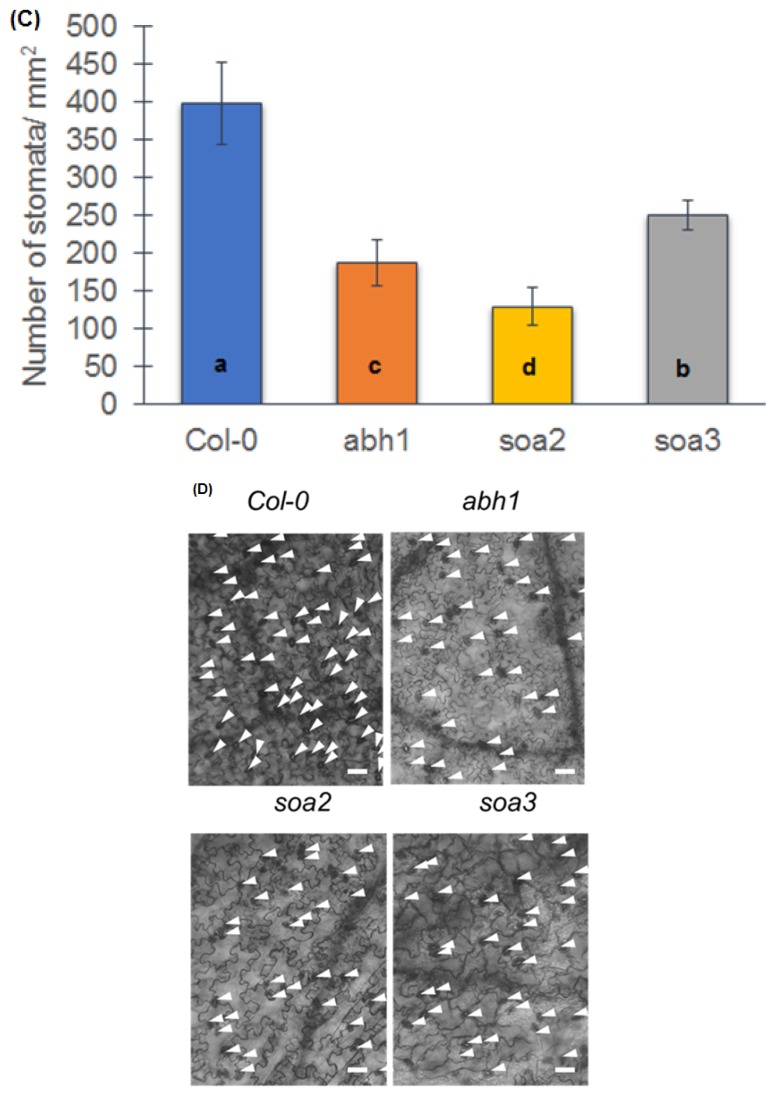
Response of Col-0, *abh1*, *soa2* and *soa3* mature plants to drought. (**A**) Relative Water Content and (**B**) Water Loss measured over 200 min; (**C**) Values represent the mean of three biological replicates. Stomatal density in rosette leaves. Values represent the mean ± SD of at least five leaves per experiment. Means followed by the same letter do not differ significantly according to Fisher’s projected LSD (*p* ≤0.05); (**D**) Stomatal density in rosette leaves, bar = 50 μm.

In order to check whether the drought-tolerant phenotype of *soa2* and *soa3* is due to the action of the stomata, like in the *abh1*, or whether it results from a decreased stomatal density, measurements of RWC (Relative Water Content) and WL (Water Loss) in detached leaves were carried out and stomatal density was estimated using a confocal laser scanning microscope. The RWC in detached leaves of *abh1* and suppressor mutants was higher than in the wild-type after 200 min and WL was much slower (Figure 7A,B). Analysis of rosette leaves examined under the confocal microscope revealed that the *abh1*, *soa2* and *soa3* had about 50% fewer stomata per mm^2^ than the wild-type (Figure 7C,D).

### 2.9. An Attempt to Identify the Suppressor Mutations Using the Candidate Genes Approach Based on Mutants Phenotype

In order to propose candidates for suppressor genes, the information from the experiments described above was used. On the basis of the phenotype traits characterized, such as response to ABA, osmotic and salt stress during seed germination and early seedling development and drought response at the mature plant stage, data-mining using the literature and sequences repositories was performed. Seven candidate genes were chosen for further analysis (Table 4). Amplified genomic sequences of the candidate genes were sequenced and the obtained sequences were aligned between *abh1* and each suppressor mutant using the CodonCode Aligner [62]. A sequence analysis revealed no mutation in any of the candidate genes analyzed.

### 2.10. Generation of Mapping Populations for Map-Based Cloning of Suppressor Genes

In order to identify the suppressor genes using a map-based cloning approach, two independent mapping populations were created. Taking into account that suppressor mutations as well as the mutation in their parental line (*abh1*) were induced in the Columbia-0 background, it was necessary to change the genetic background of *abh1* to the background of another Arabidopsis ecotype, Landsberg erecta. The original *abh1* mutant was backcrossed four times with Landsberg erecta. Then, both suppressors were crossed with *abh1* in the Landsberg erecta background. Seeds of the obtained F_1_ generation were germinated in the presence of 0.4 μM ABA and then F_1_ plants were allowed to self-pollinate in order to generate F_2_ seeds. The F_2_ population of the cross *soa3 × abh1* [Ler] was evaluated on a medium containing 0.4 μM ABA and F_2_ plants homozygous for *soa3* gene were isolated based on their ABA insensitive phenotype (Table 5).

Thirty individuals homozygous for *soa3* mutation, were used for rough mapping using 22 SSLP markers. A linkage of the *soa3* gene with *ciw4* and *nga6* markers was observed on the long arm of chromosome 3. Future work will include analysis of more F_2_ individuals (minimum 300 F_2_ individuals exhibiting suppressor phenotype) in order to increase the resolution of the mapping and to narrow the region containing a mutation by searching for recombinants between *soa3* and two flanking markers.

## 3. Discussion

In the presented study, we examined two suppressor mutants of *abh1: soa2* and *soa3*. The sensitivity to ABA during seed germination was used as a physiological marker that enabled the isolation of the suppressor mutants in the presented study. Suppressors were not only insensitive to an ABA concentration of 0.4 μM that inhibited the germination of *abh1*, but were also able to germinate in the presence of 1 μM ABA. They were not allelic and both were recessive in relation to *abh1* hypersensitivity to ABA. Further physiological and morphological analysis provided the means to distinguish between the two studied genotypes.

*soa2* mutant displayed an insensitivity to ABA both during seed germination and seedling development. When ABA was applied in a concentration of 15 μM at the seedling stage, *soa2* exhibited only a 30% root growth reduction compared to the control conditions, whereas root growth in *soa3* Col-0 and *abh1* was reduced by 70%. In addition, *soa3* displayed much higher sensitivity to ABA and NaCl during seedling growth than other genotypes studied. It can be assumed that the suppressor gene in case of *soa3* might not be the important regulator of ABA-dependent response to stress during seedling development.

Taking into account that ethylene-related mutants also display an insensitivity to high ABA concentrations in root growth [64], the impact of ACC on root growth in suppressor mutants was checked. Both *soa2* and *soa3* exhibited a 50% reduced root growth when compared to the control conditions. Together, these results suggest that the insensitivity of *soa2* to ABA during seedling development is an ABA-specific response. Similar results were observed by Beaudoin *et al*. [65] for the ABA signaling mutant—*abi1* (*ABA insensitive 1*). The effects of ethylene during seedling development seems to be independent of, or acts downstream of ABA effects at this stage, in contrast to their simultaneous actions during seed germination [65]. The role of ethylene during the seedling growth is inhibitory, whereas during seed germination it counteracts the role of ABA by promoting endosperm weakening [66].

Both suppressor mutants displayed an insensitivity to glucose and a reduced sensitivity to mannitol during seed germination. It is worth noting that *soa2* also exhibited an insensitivity to glucose and mannitol during seedling development. Under osmotic stress, in the presence of 200 mM mannitol, roots of *soa2* grew better than in control conditions, and in the presence of 400 mM mannitol, root growth was only reduced by 30%, whereas Col-0 and *abh1* displayed a 50% reduction. These results correlated with observations of seedling growth in the presence of ABA and simultaneously supported the hypothesis that the suppressor gene in *soa2* is a positive regulator of ABA signaling not only during seed germination but also during seedling development. The osmotic stress is able to induce ABA synthesis [67] and the level of synthetized ABA was not inhibitory for *soa2* root elongation since it is resistant to ABA during root growth. It can also be assumed that this ABA concentration may stimulate *soa2*’s root growth in the presence of mild osmotic stress since it is known that low ABA concentrations stimulate root growth [68]. As was mentioned above, both suppressors displayed an insensitivity to high glucose concentrations during seed germination. Xing *et al*. [63] described a similar reaction of *mkk1* (*mitogen activated protein kinase kinase 1*) and *mpk6* (*mitogen activated protein kinase 6*) mutants. Both mutants were similar to *soa2* and *soa3* when other phenotypic traits were considered, thus *MKK1* and *MPK6* were chosen for candidate genes analysis. However, none of the suppressor mutants carried a mutation in the *MKK1* and *MPK6* genes. The attempt to identify a gene carrying the mutation responsible for the mutant phenotype using the candidate genes approach was successfully used in our previously published research [55]. In the case of *soa2* and *soa3*, the candidate genes approach did not allow a suppressor gene to be identified and therefore the map-based cloning approach was used.

Suppression of *abh1* by the mutations in *soa2* and *soa3* seemed to be limited to a narrow developmental window that includes germination and early seedling development. When the drought assay was performed, both suppressor mutants were found to be drought tolerant like *abh1*. Both *abh1* and the suppressor mutants displayed the same phenotype of reduced stomata density when compared to the wild-type. It is worth noting that *soa2* displayed the most reduced number of stomata per mm^2^ and this result was correlated with the highest relative water content in leaves and the slowest water loss in the *soa2* among all of the studied genotypes. Together, these results clearly show a connection between the number of stomata and transpiration. It was demonstrated that a lower number of stomata correlates with an improved drought tolerance [69]. We can hypothesize that the ABA hypersensitivity of *abh1* stomata [53] together with a reduced stomata density ensures a drought tolerant phenotype of *abh1* and both suppressors.

To summarize, two suppressor mutants of *abh1* hypersensitivity to ABA during seed germination were characterized in regards to their response to ABA and abiotic stresses during seed germination and young seedling development and to their drought response. All observed phenotypes suggest an important role of suppressor genes in ABA-dependent abiotic stress response during seed germination or seedling development. In this study the approach of suppressor screening enabled detailed characterization of forms that potentially carry mutations in important regulators of stress signaling pathways. The suppressor screening approach has been successfully used in identification of new components or to elucidate the role of already known components of many signaling pathways as previously mentioned here. Recently, Fernández-Arbaizar *et al*. [70] reported a similar study using the suppressor screening of the jasmonate (JA)-insensitive *coronatine insensitive 1-16* (*coi1-16*) mutant and its ABA-hypersensitive phenotype during seed germination. They characterized the phenotype of several suppressor mutants in regards to abiotic stresses during seed germination, showing that the suppressor mutant screening can be successfully applied in the elucidation of the signaling pathway. Another example is the previously published *soa1* mutant carrying mutations in the *ABH1* and *ABI4* genes [55].

## 4. Experimental Section

### 4.1. Plant Material and Growth Conditions

The plant material used in this study included: insertional mutant *abh1* (*ABA hypersensitive 1*; accession Columbia-0; [40]). Col-0 used as a wild-type control, *soa2* and *soa3* (*suppressor of abh1 hypersensitivity to ABA 2* and *suppressor of abh1 hypersensitivity to ABA 3*). Suppressor mutants, *soa2* and *soa3*, were obtained after the chemical mutagenesis of *abh1* with ethyl methanesulphonate (EMS) as was described previously [55]. The same procedure of plant cultivation was applied throughout all of the experiments. First, seeds were sterilized with chlorine gas in a dessicator jar [71]. Seeds were then plated onto 0.25× Murashige-Skoog salts supplemented with 1% sucrose and solidified with 0.8% agar in the experiments where ABA was applied, or 0.5 × MS in the experiments without ABA. After 3 days at 4 °C in the dark, plates were placed in a growth chamber at 22 °C with a 16-h-light/8-h-dark cycle, 40 μmol m-2 sec-1 illumination. Four days after stratification, plantlets were transferred to Jiffy pots (Jiffy 7 Peat Pellet 42 mm, Jiffy 7C^®^, Winchester, England) and grown in a growth chamber until maturity under the conditions described above.

### 4.2. Genetic Analysis

The *soa2* and *soa3* suppressor mutants were tested for the presence of a T-DNA insertion within the *ABH1* gene using the BASTA resistance assay and PCR with specific primers to amplify the fragment containing the *ABH1* gene and T-DNA insertion. The suppressor mutants were resistant to BASTA, similar to the *abh1* mutant, whereas Col-0 did not survive the treatment. PCR amplification confirmed the presence of an insert within the CBP80 (*ABH1*) gene in the *soa2* and *soa3* mutants. An analysis led to the conclusion that the suppressor mutation is extragenic to the CBP80 (*ABH1*). The suppressor mutants displayed serrated leaves, similar to the leaves of the parental line *abh1*. An analysis of the F_2_ generation of a cross between *abh1* and Col-0 showed that leaf serration was related to the presence of a T-DNA insert within the *CBP80* (*ABH1*) gene [55].

In order to establish the mode of the inheritance of the suppressor gene, the *soa2* and *soa3* mutants were crossed with their parental line—*abh1*. Seeds of the F_1_ and F_2_ generations were screened on a medium containing 0.4 μM ABA for the selection of ABA insensitive (at the level of Col-0) and ABA hypersensitive forms according to the procedure described above.

The allelism test was performed in order to check whether the suppressor mutations were allelic or not. Seeds of F_1_ and F_2_ generations of the cross *soa2* × *soa3* and *soa3* × *soa2* were screened on a medium containing 0.4 μM ABA in the same manner as described above.

### 4.3. DNA Extraction

DNA was extracted from Arabidopsis rosette leaves using a modified C-TAB protocol [72]. Concentration and purity (A_260_/A_280_ ratio) were measured with a NanoDrop ND-1000™ spectrophotometer (ThermoScientific, Wilmington, NC, USA).

### 4.4. Seed Germination and Cotyledon Greening Assays

For each comparison, seeds of all genotypes were planted in the same plate containing an MS medium (0.25 × MS salts, 1% sucrose and 0.8% agar) with or without different concentrations of ABA (0.4; 0.6; 0.8; 1; 3) or other stress factors, such as NaCl (50; 150 and 200 mM), mannitol (100; 200; 300 mM) or glucose (4%; 6% and 4% with addition of 0.1 μM ABA). For each experiment, three biological replicates were performed, each with three technical replicates. Experiments carried out at different times and on seeds of different plants of the same genotype were considered to be biological replicates. Three independent plates in each biological replicate were considered to be technical replicates. Seeds used for these experiments were harvested and stored at the same time. Plates were chilled at 4 °C in the dark for 3 days (stratified) and moved to 22 °C with a 16-h-light/8-h-dark cycle. The percentage of seed germination was scored on the 4th day after the end of stratification. Germination was defined as the visible emergence of the radicle through the seed coat. Cotyledon greening was recorded on the 7th–10th day after the end of stratification, depending on the experiment. Cotyledon greening was defined as visible expansion and turning green of the cotyledon. The analyses were performed using a Stemi 2000-C stereoscopic microscope (Carl Zeiss, Vienna, Austria) with an attached camera (Canon, ōita, Japan). In order to document the results, AxioVision LE software (Carl Zeiss, Vienna, Austria) [73] was used. The average number of seeds analyzed in one biological replicate was 100–200.

### 4.5. Abiotic Stresses during Early Seedling Development and the Mature Plant Stage

#### 4.5.1. Root Elongation Assay in the Presence of ABA, Mannitol and NaCl

Six days after stratification, plantlets were transferred from the control medium (0.5 × MS) on the 0.5 × MS with 1% sucrose, 0.8% agar with or without ABA (1; 5; 10; 15 μM), NaCl (100; 200 mM) and mannitol (200; 400 mM). Plates were incubated vertically at 22 °C with a 16-h-light/8-h-dark cycle for 7 days. Then, photographs of seedlings and roots were taken. The analyses of root length were performed using ImageJ software (ScionImage) [74]. The average number of the seedlings of each genotype analyzed in one biological replicate was 70–100. Each experiment was replicated three times.

#### 4.5.2. Drought Treatment, Relative Water Content and Water Loss Measurements

##### 4.5.2.1. Drought Treatment

Drought treatment was applied to 3-week-old plants at the vegetative stage by withholding watering. The drought treatment lasted 26 days. Thereafter, plants were re-watered for 3 days and analysis was performed 24 h later. The drought assay was replicated three times (three biological replicates). Each experiment included 15 plants of each genotype. Chlorophyll fluorescence from leaf tissue was measured using a PocketPea Fluorometer (Hansatech^®^, Norfolk, England). The ratio of variable fluorescence to maximal fluorescence (*Fv/Fm*), representing the potential quantum yield of PSII photochemistry and PSII condition *PI*, were measured in dark-adapted leaf tissue. Leaves on intact plants were dark adapted at 22 °C for 20 min before each measurement. Five plants of each genotype were analyzed using PocketPea (Hansatech^®^, Norfolk, England) in two biological replicates.

##### 4.5.2.2. Relative Water Content and Water Loss Measurements

Relative Water Content (*RWC*, %) was calculated as the average of measurements done every 20 min during a period of 220 min according to the formula: (*F_W_* − *D_W_*)/(*T_W_* − *D_W_*) × 100% (modified) [75]. Fresh Weight (*F_W_*) was obtained by harvesting and weighing freshly detached rosette leaves every 20 min. Turgid weight (*T_W_*) was obtained by putting detached rosette leaves into an eppendorf tube with de-ionized water for 16 h at room temperature, removing excess water by wiping with absorbent paper and weighing the plant material. Dry Weight (*D_W_*) was recorded after the 24 h incubation of rosette leaves at 75 °C in a dry oven.

Water Loss (WL, %) was expressed as the percentage of the initial fresh weight of detached rosette leaves. Detached, fully expanded leaves from 4-week-old plants were incubated under the same conditions and each sample (consisting of three individual leaves) was measured in the same way as in the RWC assay. These assays were replicated three times. Each biological replicate included 5 bulks of leaves at the same developmental stage of each genotype. Three leaves are understood to comprise a bulk.

### 4.6. Observation of Stomatal Density and Preparation of Stomata Impressions

Observations were carried out using a confocal laser scanning microscope (Olympus FV1000, 488 nm wave length; Olympus, Tokyo, Japan); seedlings were treated with propidium iodide (1 mg/1 mL) before these observations. For each genotype, 10 separate fields, 0.12 mm^2^ each, of 5 leaves were observed using a 40× magnification and 10 separate fields, 0.4259 mm^2^ each, of 5 leaves were observed using a 20× magnification.

### 4.7. Chlorophyll and Carotenoids Content

Chlorophyll was extracted by boiling about 300 mg of fresh weight seedlings in 96% ethanol for 10 min at 80 °C [76]. NanoDrop Spectrophotometry (ThermoScientific, Wilmington, NC, USA) was used in the UV/VIS mode to measure the value of absorbance at 664 nm, 648.6 nm and 470 nm. Chlorophyll concentration per fresh weight was calculated as described by Lichtenthaler and Buschman [77].

### 4.8. Candidate Genes Approach

Based on the phenotype and physiological reactions of the *soa2* and *soa3* mutants to the applied stressors during germination, the candidate genes which might carry the suppressor mutation were proposed. Seven genes were chosen as candidates for sequencing and sequence analysis: *ABI3*, *ABI5*, *MYB33*, *MYB101*, *MKK1*, *MPK6* and *AREB1*. Primers were designed with Jellyfish software [78]. The primers are listed in Supplementary Table S1. The PCR profile was as follows: 94 °C-5 min; 94 °C-45 s; 60 °C-30 s, 72 °C-45 s (30 cycles) and 72 °C-5 min. PCR products were sequenced (Genomed, Warsaw, Poland) and then analyzed with a CodonCode Aligner [62].

### 4.9. Preparation of Mapping Populations

Both suppressor mutants were derived from the same ecotype—Columbia-0. In order to create a mapping population as a F_2_ progeny of the cross between the suppressor mutant and its parental line *abh1*, it was necessary to change the genetic background of one of them. *abh1* was backcrossed four times with Landsberg erecta (Ler) and then crossed with each of suppressor mutants. In the case of *soa2*, the creation of the mapping population is still in progress, whereas in the case of *soa3*, a rough mapping process has already been performed. SSLP markers were used and 30 F_2_ individuals displaying the suppressor mutant phenotype were screened in the rough mapping. Twenty-two markers were analyzed, an average 5 per one chromosome (markers used in this study were published in [79], http://carnegiedpb.stanford.edu/methods/ppsuppl.html).

## 5. Conclusions

The main objective of the presented study was to characterize the phenotypic similarities and differences between *abh1* and its suppressor mutants under a wide range of abiotic stresses. On the basis of a detailed characterization of suppressor mutants, an attempt was undertaken to identify the suppressor mutations that led to ABA insensitivity during germination. The performed physiological analyses allowed several candidates to be selected as possible suppressor genes; however, it turned out that none of them carried the mutation that was responsible for the suppressor phenotype. Rough mapping was performed for the *soa3* mutant with the result showing a linkage to two markers on the long arm of chromosome 3. Further analysis will be conducted in order to identify the suppressor genes and to confirm their impact on the suppressors’ phenotype. Identification of suppressor genes will also allow a mode of interaction between them and *CBP80* (*ABH1*) to be established.

## Supplementary Information



## Figures and Tables

**Table 1:** Genetic analysis of the inheritance mode of suppressor mutations (* *p* ≤ 0.05).

Cross	Generation	The number of analyzed seeds			χ^2^_3:1_
		Total	Col-0 sensitivity to ABA	*abh1* hypersensitivity to ABA	
***soa2*×*abh1***	F_1_	23	0	23	-
	F_2_	1057	262	795	0.025
***soa3*×*abh1***	F_1_	15	0	15	-
	F_2_	512	132	380	0.163

**Table 2:** Allelism test between s*oa2* and *soa3* mutants (^*^p ≤ 0.05).

		The number of analyzed seeds			χ^2^_11:5_
		
Cross	Generation	Total	Col-0 sensitivity to ABA	*abh1* hypersensitivity to ABA	
***soa2*×*soa3***	F_1_	25	25	-	-

	F_2_	1206	848	358	1.2

***soa3*×*soa2***	F_1_	5	5	-	-

	F_2_	1100	894	206	1.1

**Table 3:** Detailed description of genotypes and phenotypes in the cross *soa2 × soa3.* a—allele responsible for the ABA insensitivity of suppressor *soa2*; b—allele responsible for the ABA insensitivity of suppressor *soa3*; d—allele responsible for the ABA hypersensitivity of the mutant *abh1*.

*soa2* × *soa3*				

Generation		Genotype	Phenotype of ABA sensitivity	Frequency
P	*soa2*	aaBBdd	insensitive	
	*soa3*	AAbbdd	insensitive	

F1		AaBbdd	insensitive	

F2		AABBdd	hypersensitive as *abh1*	1/16
		AABbdd	hypersensitive as F1 from *soa3 × abh1*	2/16
		AaBBdd	hypersensitive as F1 from *soa2 × abh1*	2/16
		AaBbdd	insensitive as F1 from *soa2 × soa3*	4/16
		AAbbdd	insensitive as *soa3*	1/16
		Aabbdd	insensitive	2/16
		aaBBdd	insensitive as *soa2*	1/16
		aaBbdd	insensitive	2/16
		aabbdd	insensitive	1/16

The ratio of segregation of the ABA insensitive to ABA hypersensitive F2 progeny				11:5

**Table 4:** Candidate genes chosen on the basis of *soa2* and *soa3* phenotype analysis.

Gene	Mutant	Known phenotype of mutant	Reference	Phenotype of *soa2*	Phenotype of *soa3*
***ABI3***	*abi3*	ABA insensitive, reduced sensitivity to osmotic stress and glucose during seed germination	[5]	ABA insensitive, reduced sensitivity to osmotic stress and glucose during seed germination	ABA insensitive, reduced sensitivity to osmotic stress and glucose during seed germination
***ABI5***	*abi5*	ABA insensitive, reduced sensitivity to osmotic and salt stress and glucose during seed germination	[5]	ABA insensitive, reduced sensitivity to osmotic stress and glucose during seed germination	ABA insensitive, reduced sensitivity to osmotic stress and glucose during seed germination
***MYB33***	*myb33*	Insensitive to 1 μM ABA during seed germination	[60]	Insensitive to 1 μM ABA during seed germination	Insensitive to 1 μM ABA during seed germination
***MYB101***	*myb101*	Insensitive to 1 μM ABA during seed germination	[60]	Insensitive to 1 μM ABA during seed germination	Insensitive to 1 μM ABA during seed germination
***MKK1***	*mkk1*	Insensitive to ABA and glucose during seed germination	[63]	Insensitive to ABA and glucose during seed germination	Insensitive to ABA and glucose during seed germination
	*35S:mkk1*	Hypersensitive to ABA and glucose during seed germination			
***MPK6***	*mpk6*	Insensitive to glucose during seed germination	[63]	Insensitive to glucose during seed germination	Insensitive to glucose during seed germination
	*35S: mpk6*	Hypersensitive to glucose during seed germination			
***AREB1***	*areb1*	Insensitive to glucose during seed germination and drought tolerant	[36]	Insensitive to glucose during seed germination and drought tolerant	Insensitive to glucose during seed germination and drought tolerant
	*35S:areb1*	Hypersensitive to ABA			

**Table 5:** Genetic analysis of the inheritance mode of suppressor mutations based on their crosses with *abh1* [Ler] (*p* ≤ 0.05).

Cross	Generation	The number of analyzed seeds			χ^2^_3:1_
		Total	Col-0 sensitivity to ABA	*abh1* hypersensitivity to ABA	
***soa3*×*abh1*[Ler]**	F_1_	20	-	20	
	F_2_	1079	295	784	3,15
