# Supplementary material for: Towards the Identification of New Genes Involved in ABA-Dependent Abiotic Stresses Using Arabidopsis Suppressor Mutants of abh1 Hypersensitivity to ABA during Seed Germination
Source: Int J Mol Sci. 2013 Jun 27;14(7):13403–32. doi: 10.3390/ijms140713403 (PMC3742194; doi:10.3390/ijms140713403)
Supplement: Supplementary file 1 [file ijms-14-13403-s001.pdf]

Supplementary Information

Table S1. Primer sequences used in the candidate genes approach.

**ABI5F1**	**CCTCCATAACAAGAAGCGGAT**
**ABI5R1**	GGTTGTCTAGCCGCAGTCTC
**ABI5F2**	AAGAGAGGGATAGCGAACGA
**ABI5R2**	CTTTCTCCACTGGACCATCC
**ABI5F3**	AGGACAGGAGGAGGAGGGTA
**ABI5R3**	AATCACTTTCTTTGTTCACAGGG
**ABI3F1**	TGGAGTAAACCCAAACGGT
**ABI3R1**	TCAGGAAGCGTTGGGAGA
**ABI3F2**	TTGTTTCATTTCCACTTCAACG
**ABI3R2**	CATTTGCATGTCTCCACCAC
**ABI3F3**	CGAGTTCTTTGACACCTCAGC
**ABI3R3**	CGGATTCATGTTGTATCCATTG
**ABI3F4**	CACCACCACAGTCTGGTCC
**ABI3R4**	CCGGTGTTCTCGAGGAGATA
**ABI3F5**	GCAGAAAGTCTTGAAGCAAAGC
**ABI3R5**	CCGACTCGACAAGAAAAAGC
**MYB33F1**	TGGTGTTTAGGTGGGAGCTTG
**MYB33R1**	CTTCGAGGAGGGGATTGTTG
**MYB33F2**	GCAGCTGGGGAGTTGTGAAT
**MYB33R2**	CACTTGGTGGAGTTTCATCTGC
**MYB33F3**	GCTGCGCTTCAACTATTCCA
**MYB33R3**	CGGCTGTGATCAAAAGGCTC
**MYB101F1**	TTCTTCCTCTCCTTGATCGGA
**MYB101R1**	TTGGAACTGAAAGGAAGTTAAATCA
**MYB101F2**	TGGAACACGAGGATGAAGAGA
**MYB101R2**	TGTGCTTACCCAATGATGAATGA
**MYB101F3**	CTTGTCTCGAGGCGGACTCT
**MYB101R3**	GCACATCCCAAGTTGAAAACA
**MKK1_F1**	CGACGGTTCGTCATTCTTC
**MKK1_R1**	AATCGCCCGACATGTTGA
**MKK1_F2**	CTCAACAGTTTTTCGCTCTTAAGG
**MKK1_R2**	TACTCCATCCTTTCTTGTGTTCTG
**MKK1_F3**	TATTTGGAGCTTGGGACTGG
**MKK1R3**	AAGTATATCCACCACTAGCAGAGAAA
**MPK6F1**	GACCCCAAAACCAACGAATA
**MPK6R1**	CCTAAACAATTTTGCATCAGC
**MPK6F2**	CTGCTTCGTCACATGGATCA
**MPK6R2**	AGGGAAGAGTGGCTTACGGT
**MPK6F3**	CTGCAGCTATCGATGTTTGG
**MPK6R3**	CGCCAAACCCTTATCTCATC
**AREB1F1**	GAGAGACACTTGGCTCACCA
**AREB1R1**	TTGCTGCCTCTGACTCTGAC
**AREB1F2**	GGAAGTAGTGGAGTAGGGGGA
**AREB1R2**	TTGCGAGATTCATTCAGCC
**AREB1F3**	CAAAGCGTAAAGTGTCTTGTGTG
**AREB1R3**	CAGAAACTAAGTATGATGGCATTGA
